# Effect of primary copper metabolism disturbance on elemental, protein, and lipid composition of the organs in Jackson toxic milk mouse

**DOI:** 10.1007/s10534-024-00640-y

**Published:** 2024-10-04

**Authors:** Krzysztof Hadrian, Magdalena Szczerbowska-Boruchowska, Artur Surówka, Olga Ciepiela, Tomasz Litwin, Adam Przybyłkowski

**Affiliations:** 1 Department of Gastroenterology and Internal Medicine, Medical University of Warsaw, Warsaw, Poland; 2 Department of Medical Physics and Biophysics, AGH University of Science and Technology, Cracow, Poland; 3 Department of Laboratory Medicine, Medical University of Warsaw, Warsaw, Poland; 4 Second Department of Neurology, Institute of Psychiatry and Neurology, Warsaw, Poland

**Keywords:** Wilson’s disease, Toxic milk mouse, Neurodegeneration, Copper metabolism, Neurofilament

## Abstract

Toxic milk (txJ) is an autosomal recessive mutation in the *Atp7b* gene in the C3H/HeJ strain, observed at The Jackson Laboratory in Maine, USA. TxJ mice exhibit symptoms similar to those of human Wilson’s disease (WD). The study aimed to verify organ involvement in a mouse model of WD. TxJ mice and control animals were sacrificed at 2, 4, 8, and 14 months of age. Total X-ray Fluorescence Spectroscopy (TXRF) was used to determine the elemental concentration in organs. Tissue chemical composition was measured by Fourier Transform Infrared Spectroscopy (FTIR). Additionally, hybrid mapping of FTIR and microXRF was performed. Elevated concentrations of Cu were observed in the liver, striatum, eye, heart, and duodenum of txJ mice across age groups. In the striatum of the oldest txJ mice, there was lower lipid content and a higher fraction of saturated fats. The secondary structure of striatum proteins was disturbed in txJ mice. In the livers of txJ mice, higher concentrations of saturated fats and disturbances in the secondary structure of proteins were observed. The concentration of neurofilaments was significantly higher in txJ serum. The distribution of Cu deposits in brains was uniform with no prevalence in any anatomic structure in either group, but significant protein structure changes were observed exclusively in the striatum of txJ. In this txJ animal model of WD, pathologic copper accumulation occurs in the duodenum, heart, and eye tissues. Increased copper concentration in the liver and brain results in increased saturated fat content and disturbances in secondary protein structure, leading to hepatic injury and neurodegeneration.

## Introduction

Wilson’s disease (WD) is a genetic condition that affects the body’s ability to process copper (Wu et al. 2015). The gene responsible for this disorder is located on human chromosome 13 (Beyzaei et al. 2024). It encodes a protein called ATP7B, which functions as a P-type ATPase responsible for copper transport. When ATP7B malfunctions, copper accumulates in the liver, brain, and other organs, leading to organ damage and a range of symptoms affecting the liver and nervous system (Ovchinnikova et al. 2024). Similar copper metabolism disorders have been observed in animals, such as Long Evans Cinnamon (LEC) rats and toxic milk mice, both of which have mutations in the rodent counterpart of the *Atp7b* gene (Medici and Huster 2017; Reed et al. 2018).

Toxic milk (tx) is an autosomal recessive mutation found in the *Atp7b* gene in C57BL/6 J mice. This mutation emerged spontaneously in the laboratory of the University of Massachusetts and was studied by Rauch (1983). Another recessive mutation in the murine *Atp7b* gene was discovered in the C3H/HeJ strain at The Jackson Laboratory in Maine, USA (Coronado et al. 2001). This mutation, known as txJ, is a point mutation at position 2135 in exon 8, resulting in a missense mutation in ATPase, where glycine is replaced by aspartic acid (G712D). Although this specific mutation has not been observed in WD patients, Gly710 is a hotspot for congruent, well-described human mutations (Chang and Hahn 2017). TxJ mice exhibit symptoms similar to those of human WD and show copper accumulation in various organs. In both WD patients and toxic milk mice, ATP7B dysfunction leads to disturbances in copper hepatic biliary excretion and incomplete synthesis of the cuproenzyme ceruloplasmin. As a result, serum ceruloplasmin activity is low due to its rapid degradation in affected humans and animals (Lucena-Valera et al. 2023).

Liver involvement in toxic milk mice, similar to humans, is characterized by steatosis (accumulation of fat in the liver), mild inflammation, and gross nodularity, which resembles the pathology observed in humans with WD (Theophilos et al. 1996; Schroeder et al. 2021). Besides liver disease, approximately 40% of WD patients also experience neurological symptoms (Poujois et al. 2017). The typical brain abnormalities in WD include degeneration of the putamen and globus pallidus, as well as lesions in the caudate, thalamus, brainstem, and cerebellum (Shribman et al. 2021a). The neurological symptoms in WD are classified into different syndromes, such as akinetic-rigid syndrome (similar to Parkinson’s disease), pseudosclerosis with tremors as the main symptom, ataxia, and dystonic syndrome (Dusek et al. 2019a). There have been only a few reports of neuronal injury and neurological symptoms in the txJ strain (Przybyłkowski et al. 2013; Bronson and Davisson 1995; Zhou et al. 2019; Terwel et al. 2011).

Recent studies on animal WD models have focused on liver and brain involvement, but research on metal deposition in other organs, such as the heart, eye, and intestine, is exceptionally rare. These organs were analyzed and summarized in this project (Peng et al. 2012a, 2012b; Hou et al. 2006).

Despite significant data on brain copper accumulation in previous studies and hypotheses about how copper enters the brain, there is a lack of information about its later allocation and direct influence on biochemical changes. The authors aim to determine the manner of copper distribution after ions enter the txJ central nervous system and their impact on brain protein and lipid status.

The main aim of the study was to verify organ involvement in txJ mice.

## Materials and methods

### Animals

The experiments were conducted in strict compliance with the European Communities Council Directive of November 24th, 1986 (86/609/EEC) and the Animal (Scientific Procedures) Act of the Republic of Poland. The C3HeB/FeJAtp7btx-J/J mice, as well as the control C3HeB/FeJ mice, were obtained from The Jackson Laboratory in Bar Harbor, ME, USA, and were bred locally. Due to their unique needs, the txJ mouse pups required fostering by healthy dams to ensure their survival. Within five days of birth, they were removed from their mothers and placed with lactating BALB/cByJ females for feeding and fostering. The BALB/cByJ mice were chosen for cross-fostering as recommended by The Jackson Laboratory due to their good reproductive performance and minimal aggression.

Following weaning, the mice were housed in a controlled environment with a normal light/dark cycle of 12 h each. The temperature and humidity were maintained at appropriate levels. Mice from the same litter and of the same sex were housed together in groups of 1–5 per cage. They were provided with ad libitum access to food and water through elongated spout bottles. A total of 61 txJ mice and 81 control mice were included in the project. The experimental groups consisted of 3–14 animals, maintaining a gender ratio of 1:1 (if possible). The experiments were conducted on mice at 2, 4, 8, and 14 months of age (Table 1).

**Table 1:** Animals included in the project

Age [months]	txJ *n* = 61		Controls *n* = 81	
	Male	Female	Male	Female
2	9	9	9	8
4	9	9	9	9
8	8	9	9	9
14	3	5	14	14

### Activity of ceruloplasmin

The concentration of ceruloplasmin in the serum of each individual was determined using the method described by Schosinsky et al. (1974). The WPA CO7500 colorimeter (Biochrom, UK) was used for the measurements. The activity was measured in duplicate after a 60-min incubation with o-dianisidine at a wavelength of 540 nm.

### Liver function tests

Aspartate aminotransferase (AST), alanine aminotransferase (ALT), bilirubin, and albumin were tested using standard protocols with a Cobas 8000CC analyzer (Roche, Switzerland), while alkaline phosphatase (ALP) was tested using a Cobas c 701/702 analyzer (Roche Diagnostics, USA).

### Neurofilament protein (low molecular weight)—(Nf-L)

Nf-L is a lightweight protein of the cytoskeleton that is released following neuroaxonal damage. Nf-L levels can be assessed in both cerebrospinal fluid (CSF) and serum, and they are significantly elevated in various neurological disorders involving considerable neuroaxonal damage. As a result, measuring serum Nf-L concentration has been recognized as an important biomarker in basic and clinical research (Wang et al. 2020a; Shribman et al. 2021b). The measurement of neurofilament protein concentration in serum was conducted using the colorimetric ELISA Mouse Nf-L ELISA Kit (Novus Biologicals, USA) according to the manufacturer’s instructions.

### TXRF

To determine the elemental content (S, Cl, K, Ca, Cr, Fe, Mn, Ni, Cu, Zn, Ga, Se, Br, Rb, Sr) in the striatum, liver, heart, duodenum, and eyeball, TXRF was employed. In our study, we focused on tissues and organs affected by Wilson’s disease, as described in humans (Dzieżyc-Jaworska et al. 2019). The advantage of the method used is the minimal distortion in the final result caused by background noise, thanks to the examination technique and the geometry of the radiation beam barely touching the examined sample. Another benefit of this method is its high sensitivity in measuring trace elements with an accuracy of up to 1 part per billion (ppb), which is essential in the medical biochemical analysis of tissues of biological origin. It allows for the simultaneous analysis of multiple elements in a single measurement, ensuring an efficient assessment of changes in the elemental composition of the examined tissues and organs.

Appropriate tissue samples were prepared from the heart, liver, duodenum, and the entire eyeball. Immediately after thawing, the striata were prepared from the brain. Samples were placed in acid-resistant vessels (Anton Parr), and nitric acid (V) (supra-pure quality) was added (0.5–0.6 mL) along with perhydrol (50 µL). The vessels were tightly closed and placed in an oven for 8 h at 200 °C. After digestion, an internal standard of 10 μL of a Ga solution (1000 mg/L, Merck) was added to the samples for subsequent quantitative analysis. The final concentration of Ga ranged from 40 to 95 mg/L, depending on the tissue weight. Measurements were performed on Menzel Glaser Superfrost slides (Thermo Scientific, Germany) in triplicate on the TXRF spectrometer, Nanohunter II (Rigaku, Japan). A molybdenum X-ray lamp was used at an energy of 55 kV and a current of 12 mA, with a measurement time of 1000 s.

### FTIR

For the determination of chemical composition, FTIR was employed. The FTIR method allows for a simple, rapid, cost-efficient, and nondestructive analysis of biomolecules (Rohman et al. 2019). A total of 285 samples of frontal brain and liver cross-sections were prepared. The cutting thickness was set to 10 µm using a cryostat CM1860UV (Leica, Germany), low-profile interchangeable blades DB80LX (Leica, Germany), and a cryogenic embedding matrix OCT (Leica, Germany). The sections were applied to circular calcium fluoride slides with a diameter of 25 mm and a thickness of 2 mm (Crystan, UK). The samples were dehydrated at − 80 °C using a vacuum method. Measurements were carried out in the spectral range of 4000–1000 nm, with a resolution of 4 nm, using a photodiode InGaAs detector, 128 scans for background measurement, 32 scans for sample measurement, Happ-Genzel apodization, and a Magna-IR560 spectrometer (Thermo Nicolet, USA) in FTIR transmission mode.

All samples for FTIR and Hybrid Mapping were prepared at − 20 °C using a cryostat CM1860UV (Leica, Germany), low-profile blades DB80LX (Leica, Germany), and cryogenic embedding matrix OCT (Leica, Germany).

The FTIR data processing was conducted using in-house Python 2.7 code, utilizing the numpy, pandas, and matplotlib packages. Our code solutions were based on simple peak area integration or peak intensity (peak height) calculation with basic linear baseline correction. To facilitate the interpretation of results, distinctive ratios were defined, allowing for semi-quantitative analysis. These included the lipid/protein ratio (L/P), carbonyl/lipid ratio (Carb/L), lipid unsaturation parameter (L_Unsat_), fatty acid chain length parameter (FACL), α/β protein secondary structure ratio (P_α_/P_β_), and the fraction of β-sheet and β-turn forms contributing to all amide I forms (P_%_ β). L_Unsat_ is a parameter highlighting lipid structure changes in tissues. It is computed as the ratio of the integrated area of the CH olefinic bonding in unsaturated lipid chains to the integrated area of the –CH_3_ antisymmetric stretching characteristic of lipid side chains. FACL, which describes the relative lengths of hydrocarbon fatty acyl chains in lipids, was computed as the ratio of the integrated area of the –CH_2_ antisymmetric stretching (representing the –CH_2_– group occurring along the fatty acyl chain) to the integrated area of the –CH_3_ stretching (representing the terminal –CH_3_ group on the fatty acyl chain). A higher FACL value indicates longer fatty acyl chains.

Carb/L is a parameter representing the asymmetry of molecular distribution, providing information about lipid esters. It was computed as the ratio of the integrated area of the triglyceride C = O carbonyl stretching to the integrated area of C–H stretching. L/P reveals molecular asymmetry in the examined structures and was computed as the ratio of the integrated area of the C–H stretching to the integrated area of the amide I region.

Protein secondary structure aberrations, in terms of biochemical changes in the conformation of β-sheets and α-helices, were determined by calculating both the α-helix to β-sheet ratio and the percentage fraction of β-turns and β-sheets.

In FTIR measurements, a “Fingerprint” refers to a specific region of the infrared spectrum (typically 1500–400 cm^−1^) that contains unique absorption bands characteristic of particular substances. This region includes complex vibrations that provide a distinctive spectral pattern for the material being analyzed. The fingerprint region is often used to identify and differentiate between various chemical compounds, as different substances will have unique absorption patterns in this area due to their molecular structure. Comparing the fingerprint spectra of an unknown sample to reference spectra allows for the identification of the chemical composition and functional groups present. In the performed analysis, due to the complexity of the samples, the “Fingerprint” represents the total molecular content in the sample.

### *Hybrid (microXRF* + *FTIR) mapping*

The authors aimed to compare maps of brain essential element distribution (microXRF) with those of chemical composition (FTIR). Samples of frontal brain cross-sections were prepared with a cutting thickness of 20 µm. The sections were applied to ultralene film (SPEX SamplePrep, USA) with a diameter of 64 mm and a thickness of 0.16 mm, and placed in plexiglass holders. The samples were dehydrated at − 80 °C. Measurements were conducted using the M4 Tornado microspectrometer (Bruker, USA).

### Statistics

All statistical calculations were performed using Statistica ver. 13.3 (StatSoft, Poland). Given the small size of each animal group and after testing data for normality using the Shapiro–Wilk test, the authors decided to use the Mann–Whitney *U* test for all calculations in this project. The significance level was set at *p*-value < 0.05.

## Results

### Metal composition

#### Liver

**Fig. 1:**
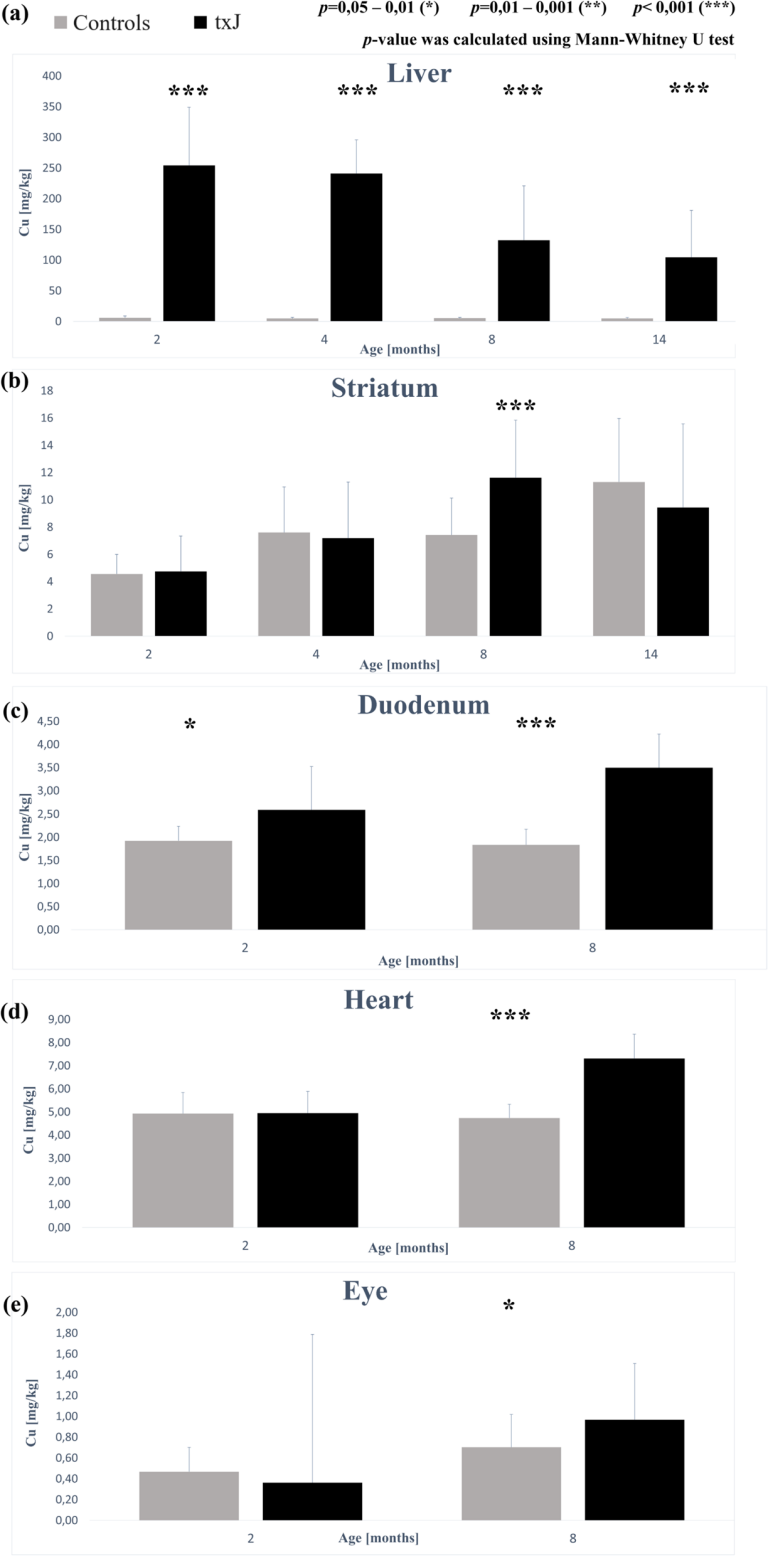
The effect of congenital copper metabolism disorder on its content in various organs (**A**–**E**) during ontogenesis (according to TXRF analysis). **A** Concentration of Cu [mg/kg] in the liver in age groups [months]. **B** Concentration of Cu [mg/kg] in the striatum in age groups [months]. **C** Concentration of Cu [mg/kg] in the duodenum in age groups [months]. **D** Concentration of Cu [mg/kg] in the heart in age groups [months]. **E** Concentration of Cu [mg/kg] in the eye in age groups [months]

The concentration of copper was significantly higher in all age groups in txJ mice. The Cu concentration was highest at 2 months and decreased progressively with age (Fig. 1A). The zinc concentration in the txJ liver was also highest at 2 months and tended to decline in subsequent measurements, with significant differences noted at 2, 4, and 14 months. In the control group, the zinc concentration remained stable across all measurements (Table 2).

**Table 2:** Liver elemental composition (TXRF) [mg/kg]

Age (Months)	2					4					8					14				
Element	Controls		Cases		*p*	Controls		Cases		*p*	Controls		cases		*p*	Controls		Cases		*p*
	Median	IQR	Median	Median		Median	IQR	Median	IQR		Median	IQR	Median	IQR		Median	IQR	Median	IQR	
Cr	0.74	0.28	0.97	0.97	0.05	0.76	0.35	0.77	0.20	0.73	1.03	0.34	0.98	0.40	0.55	0.95	0.56	1.22	1.50	0.31
Fe	145.22	65.02	186.81	186.81	0.05	158.88	96.47	171.57	37.93	0.85	217.56	85.18	160.43	95.08	0.08	189.63	114.12	192.34	152.86	0.19
Zn	30.92	5.94	72.23	72.23	< 0.001	29.77	4.60	59.99	5.96	< 0.001	33.17	6.75	33.72	15.84	0.23	27.77	7.38	33.22	13.72	0.04
**Ga**^**a**^	248.18	75.33	128.20	128.20	< 0.001	257.70	144.05	235.67	36.32	0.42	255.80	135.00	220.32	49.98	0.02	255.60	100.70	264.60	168.30	0.84

*p-*value was calculated using the Mann–Whitney U test

^a^Internal standard

#### Brain

**Map 1:**
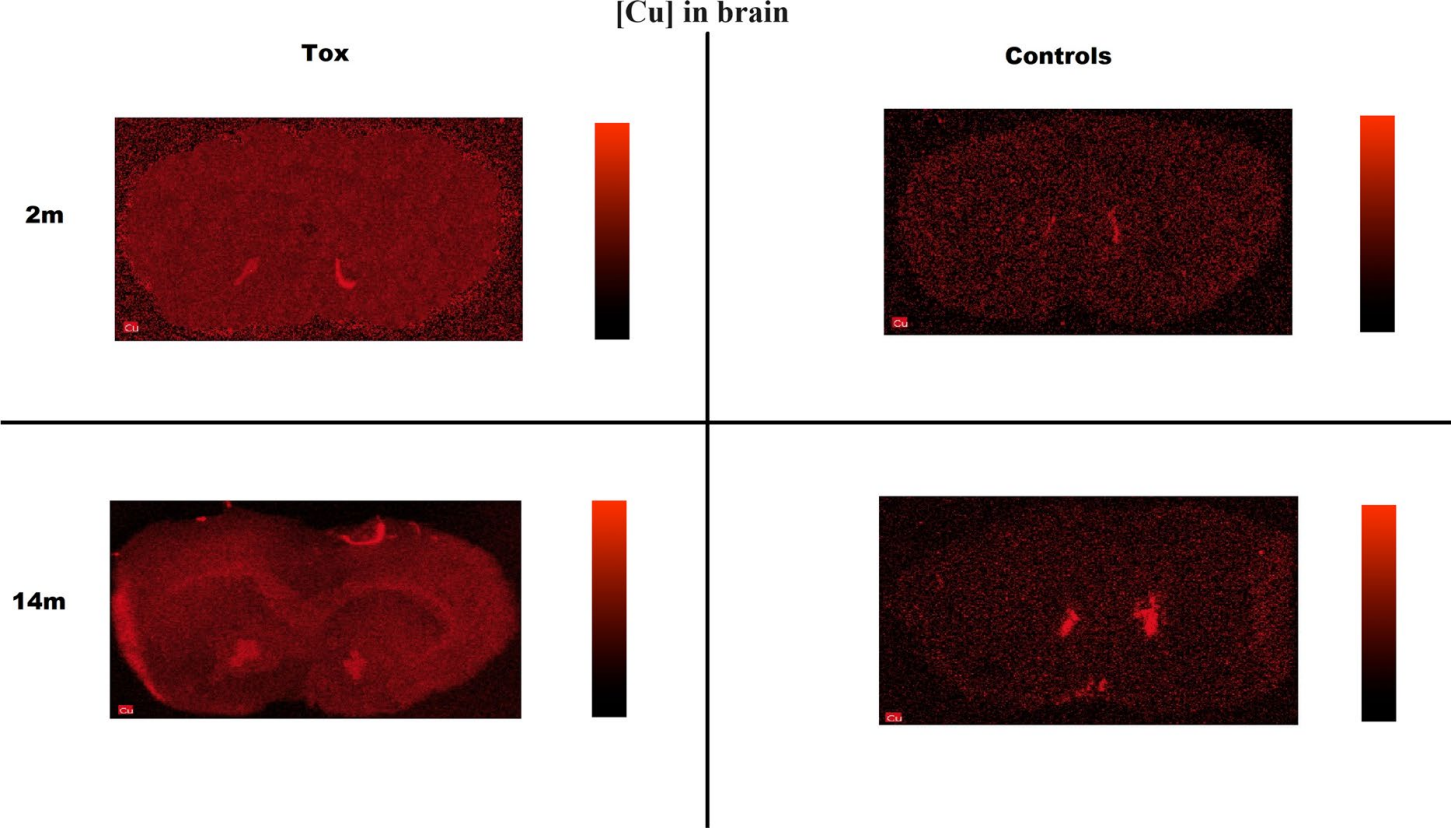
MicroXRF-Cu distribution in brain coronal cross-sections

The concentration of copper in the striatum at 2 and 4 months was comparable between the txJ and control groups. Clear differences in Cu concentration appeared in txJ mice at 8 months. At 14 months, the concentration remained higher in the txJ group, although this difference was not statistically significant (Fig. 1B). In hybrid mapping, the concentration of copper was higher in txJ animals, but the distribution of copper deposits was uniform, with no visible prevalence in any specific brain structure, similar in both groups (Map 1).

The iron concentration was initially higher in the txJ group. By the 2nd measurement, a significant decrease was noted, while the control group showed a uniform increase over time. Iron concentrations at 8 and 14 months were similar between the groups. Zinc levels in the striatum of txJ mice showed the greatest differences at 2 months. In the next measurement, the concentration increased but then decreased uniformly in the subsequent two measurements. Zinc concentration in the control group followed a comparable trend (Table 3).

**Table 3:** Striatum elemental composition (TXRF) [mg/kg]

Age (Months)	2					4					8					14				
Element	Controls		Cases		*p*	Controls		Cases		*p*	Controls		Cases		*p*	Controls		Cases		*p*
	Median	IQR	Median	IQR		Median	IQR	Median	IQR		median	IQR	Median	IQR		Median	IQR	Median	IQR	
Cr	0.86	1.25	1.12	2.24	0.14	0.90	0.86	0.77	1.10	0.49	0.77	1.10	0.55	0.39	0.07	0.51	0.96	1.73	0.97	< 0.001
Fe	21.89	12.49	30.76	20.30	0.13	33.50	14.23	27.03	13.05	0.04	36.76	9.70	37.74	19.24	0.90	40.28	17.50	40.33	16.37	0.87
Zn	12.96	3.00	15.20	4.19	0.01	15.76	3.38	16.35	5.80	0.99	14.70	5.30	15.76	4.89	0.06	14.67	2.33	13.48	4.81	0.35
**Ga**^**a**^	0.17	0.05	0.20	0.05	0.02	0.22	0.08	0.20	0.09	0.13	0.22	0.07	0.24	0.09	0.26	0.23	0.10	0.20	0.06	0.15

*p-*value was calculated using the Mann–Whitney U test

^a^Internal standard

#### Duodenum

The copper concentration in the duodenum was higher in the txJ group at 2 and 8 months and showed an increasing trend. In the control group, the opposite trend was observed. The differences were statistically significant at both time points (Fig. 1C). The chromium concentration in the txJ duodenal wall was lower at both time points, though both groups exhibited an increasing trend. The iron concentration in both groups reached comparable values and maintained a similar increasing trend. Zinc concentration was higher in the txJ group at the first measurement but decreased over time, reaching a lower value than the control group by 8 months. The control group maintained relatively stable zinc levels in both measurements (Table 4).

**Table 4:** Duodenum elemental composition (TXRF) [mg/kg]

Age (Months)	2					8				
Element	Controls		Cases		*p*	Controls		Cases		*p*
	Median	IQR	Median	IQR		Median	IQR	Median	IQR	
Cr	0.55	0.12	0.25	0.14	< 0.001	0.34	0.19	0.38	0.27	0.56
Fe	69.02	32.17	67.84	29.86	0.55	82.95	19.93	83.43	15.99	0.81
Zn	23.85	2.31	25.56	3.17	0.14	24.06	2.59	22.24	3.08	0.11
**Ga**^**a**^	173.35	47.7	207	72.8	0.08	134.5	26.8	170.35	83.75	0.002

*p-*value was calculated using the Mann–Whitney U test

^a^Internal standard

#### Heart

At 2 months, the copper concentration in the heart was comparable between both groups. In the txJ group, a higher copper concentration was observed at 8 months, while in the control group, the concentration slightly decreased compared to the first measurement, resulting in statistically significant differences at the final measurement (Fig. 1D). The chromium concentration at 2 months was significantly higher in the control group, with a decreasing trend observed over time. In contrast, the txJ group exhibited the opposite trend, surpassing the control group’s Cr concentration at 8 months. The iron concentration in the heart at 2 months was lower in the txJ group and increased over time, surpassing the control group’s concentration at 8 months, which remained relatively stable. Zinc concentration was higher in the txJ group at both time points, with a decreasing trend observed in both groups. By the final measurement, the zinc concentration in txJ mice was significantly higher (Table 5).

**Table 5:** Heart elemental composition (TXRF) [mg/kg]

Age (Months)	2					8				
Element	Controls		Cases		*p*	Controls		Cases		*p*
	median	IQR	median	IQR		median	IQR	median	IQR	
Cr	0.52	0.23	0.33	0.14	0.02	0.37	0.19	0.43	0.25	0.67
Fe	144.30	44.70	118.26	57.89	0.15	146.99	30.48	165.55	62.87	0.63
Zn	15.63	1.11	16.03	2.51	0.80	14.47	1.29	15.06	4.63	0.03
**Ga**^**a**^	164.60	41.30	194.00	22.40	< 0.001	138.00	43.60	150.90	37.90	0.16

*p-*value was calculated using the Mann–Whitney U test

^a^Internal standard

#### Eye

At 2 months, the copper concentration was lower in the txJ group. Both groups experienced an increase in copper concentration over time, and by 8 months, the copper concentration in the txJ group was statistically significantly higher than in the control group (Fig. 1E). The concentration of chromium in the eye decreased in the txJ group, while the control group showed the opposite trend. The concentration of iron increased over time in both groups. At 2 months, the iron concentration was lower in the txJ group, but by 8 months, it surpassed the control group. The zinc concentration in the txJ group increased over time, showing a significant difference compared to the control group, where the concentration at 8 months was lower than in the first measurement (Table 6).

**Table 6:** Eye elemental composition (TXRF) [mg/kg]

Age (Months)	2					8				
Element	Controls		Cases		*p*	Controls		Cases		*p*
	Median	IQR	Median	IQR		Median	IQR	Median	IQR	
Cr	0.30	0.47	0.39	0.15	0.45	0.42	0.73	0.31	0.53	0.60
Fe	12.58	3.72	11.87	6.86	0.81	12.75	8.53	13.13	7.51	0.76
Zn	4.33	0.95	4.70	1.01	0.20	4.23	1.09	5.08	0.76	0.01
**Ga**^**a**^	435.75	54.50	438.35	24.30	0.61	357.00	49.07	354.60	19.50	0.62

*p-*value was calculated using the Mann–Whitney U test

^a^Internal standard

### Serum tests

#### Ceruloplasmin

The median serum ceruloplasmin concentration was significantly lower in the txJ group at all time points. Serum albumin concentration was also lower in the txJ group at all time points, with statistical significance achieved in the 14-month-old mice. Liver function test activities were elevated in the txJ group, with significant differences mainly observed at 4 and 8 months (Table 7).

**Table 7:** Serum biochemical analysis

Age (Months)	2					4					8					14				
Parameter	Controls		Cases		*p*	Controls		Cases		*p*	Controls		Cases		*p*	Controls		Cases		*P*
	Median	IQR	Median	IQR		Median	IQR	Median	IQR		Median	IQR	Median	IQR		Median	IQR	Median	IQR	
Ceruloplasmin [IU/l]	0.32	0.05	0.09	0.05	< 0.001	0.36	0.07	0.14	0.04	< 0.001	0.33	0.05	0.19	0.08	< 0.001	0.43	0.35	0.15	0.09	< 0.001
Nf-L [ng/ml]	0.24	0.13	0.27	0.15	0.27	0.18	0.12	0.23	0.06	0.01	0.24	0.13	0.29	0.13	0.27	0.18	0.15	0.28	0.16	0.04
ALT [IU/l]	70.00	38.00	77.50	59.50	0.59	104.00	67.00	186.50	107.50	0.01	100.00	62.00	420.00	330.00	< 0.001	74.00	69.00	119.00	62.00	0.05
ALP [IU/l]	179.50	40.00	218.50	63.50	0.01	100.00	20.00	187.00	42.50	< 0.001	104.50	36.50	253.00	42.00	< 0.001	120.00	139.00	186.50	85.50	0.41
Bilirubin [mg/dl]	0.10	0.10	0.15	1.90	0.48	0.10	0.30	2.20	2.55	0.06	0.10	0.00	0.20	0.60	0.01	2.00	1.10	1.55	2.00	0.24
Albumin [g/dl]	2.95	1.80	3.01	1.04	0.90	3.00	1.51	2.57	1.46	0.21	3.00	1.15	2.20	1.00	0.07	3.33	0.34	3.03	0.77	0.03

*p-*value was calculated using the Mann–Whitney U test

#### Alanine transaminase

ALT activity in the serum at 2 months was comparable between both groups, with no significant difference. However, in the next two age groups, significantly higher ALT activity was observed in the txJ group, peaking at 8 months. At 14 months, ALT activity in the txJ group was notably lower than at 8 months but remained significantly higher than in the control group. ALT serum activity remained comparable across all measurements in the control group.

#### Neurofilament protein (low molecular weight) concentration

The serum concentration of neurofilament protein differed significantly between the groups, with the most statistically significant differences observed at 4 and 14 months (Table 7).

### Chemical composition

#### Liver

Aberrations in the secondary structure of liver proteins were observed across the entire txJ population. Statistically significant differences were noted at 4 and 8 months. Protein structure disturbances at these same points were confirmed by measurements of the Amide I max position in liver samples. A significant imbalance between lipid and protein concentrations in the txJ liver was observed, with the most pronounced differences in the 4-month-old group. Additionally, disturbances in ester concentration in txJ livers were noted, with significant differences at 2, 4, and 8 months As with the striatum, the concentration of saturated lipids in the txJ liver was higher at all time points and increased with age (Table 8).

**Table 8:** FTIR analysis of the chemical structure of the liver

Age (Months)	2					4					8					14				
Parameter	Controls		Cases		*p*	Controls		Cases		*p*	Controls		Cases		*p*	Controls		Cases		*p*
	Median	IQR	Median	IQR		Median	IQR	Median	IQR		Median	IQR	Median	IQR		Median	IQR	Median	IQR	
Amide I	1.82	1.67	1.28	1.24	0.56	1.08	1.77	1.84	1.83	0.05	1.90	2.72	2.51	1.67	0.09	1.45	2.39	1.71	2.59	0.62
Amide I max.-position	1646.43	17.84	1640.40	22.18	1.00	1646.43	28.44	1651.97	4.82	0.01	1647.87	18.80	1652.21	0.48	0.04	1651.73	17.84	1652.21	5.79	0.43
Fingerprint	13.37	12.98	12.98	7.13	0.68	8.12	14.11	14.37	8.05	0.02	13.55	17.81	16.88	7.95	0.70	12.24	11.65	15.21	13.79	0.72
Esters	0.19	0.10	0.34	0.26	0.01	0.27	0.25	0.11	0.08	< 0.001	0.21	0.23	0.11	0.10	0.02	0.12	0.27	0.08	0.09	0.24
Protein beta/alpha	1.08	0.18	1.07	0.18	0.83	1.03	0.30	0.95	0.16	< 0.001	1.02	0.27	0.93	0.07	< 0.001	1.02	0.27	1.02	0.13	0.78
Lipid/protein	0.10	0.32	0.00	0.14	0.24	0.37	0.59	0.09	0.26	0.03	0.28	0.45	0.22	0.21	0.55	0.23	0.41	0.15	0.24	0.47
Lipid saturation	0.09	0.07	0.34	0.26	0.01	0.11	0.05	0.21	0.08	< 0.001	0.11	0.13	0.27	0.10	< 0.001	0.18	0.07	0.32	0.09	0.002

*p-*value was calculated using the Mann–Whitney U test

#### Brain

Lipid saturation between 2 and 8 months was comparable in both groups. However, at 14 months, lipid saturation was significantly higher in the striata of txJ mice. The relative lipid concentration was initially similar in both groups and increased over time—up to 8 months in the txJ group and up to 4 months in the control group, after which it remained stable in the control group between 4 and 8 months. In the experimental group, lipid concentration showed a declining trend after 8 months, reaching a significantly lower value by 14 months compared to the control group.

Differences in striatum protein structure between the groups were first observed at 8 months, where the ratio of beta to alpha structure was significantly lower in the txJ population. This trend reversed at 14 months when the ratio of beta to alpha forms was significantly higher in the txJ population (Table 9).

**Table 9:** FTIR analysis of the chemical structure of the striatum

Age (Months)	2					4					8					14				
Parameter	Controls		Cases		*p*	Controls		Cases		*p*	Controls		Cases		*p*	Controls		Cases		*p*
	Median	IQR	Median	IQR		Median	IQR	Median	IQR		Median	IQR	Median	IQR		Median	IQR	Median	IQR	
Amide I	1.68	1.97	0.78	1.49	0.04	1.39	1.51	0.89	1.55	0.84	1.75	1.08	1.23	1.46	0.95	1.15	1.98	1.11	1.56	0.85
Amide I max.-position	1652.21	5.30	1652.21	7.71	0.26	1652.21	2.89	1652.94	1.93	0.68	1652.21	2.41	1654.14	2.41	0.15	1653.90	2.41	1650.29	4.34	0.02
Protein beta/alpha	0.97	0.24	1.05	0.46	0.66	0.71	0.16	0.68	0.34	0.80	0.75	0.21	0.67	0.11	0.02	0.68	0.14	0.96	0.13	< 0.001
Lipid relative	0.09	0.04	0.08	0.09	0.76	0.17	0.06	0.17	0.08	0.76	0.16	0.09	0.20	0.07	0.07	0.18	0.08	0.13	0.04	0.02
Protein relative	0.11	0.09	0.06	0.13	0.22	0.20	0.06	0.20	0.07	0.91	0.16	0.05	0.21	0.10	0.002	0.18	0.08	0.14	0.04	0.06
Lipid saturation	0.24	0.08	0.24	0.25	0.75	0.18	0.06	0.19	0.05	0.94	0.19	0.05	0.18	0.02	0.06	0.18	0.02	0.24	0.03	< 0.001

*p-*value was calculated using the Mann–Whitney U test

#### Hybrid microXRF/FTIR mapping

**Map 2:**
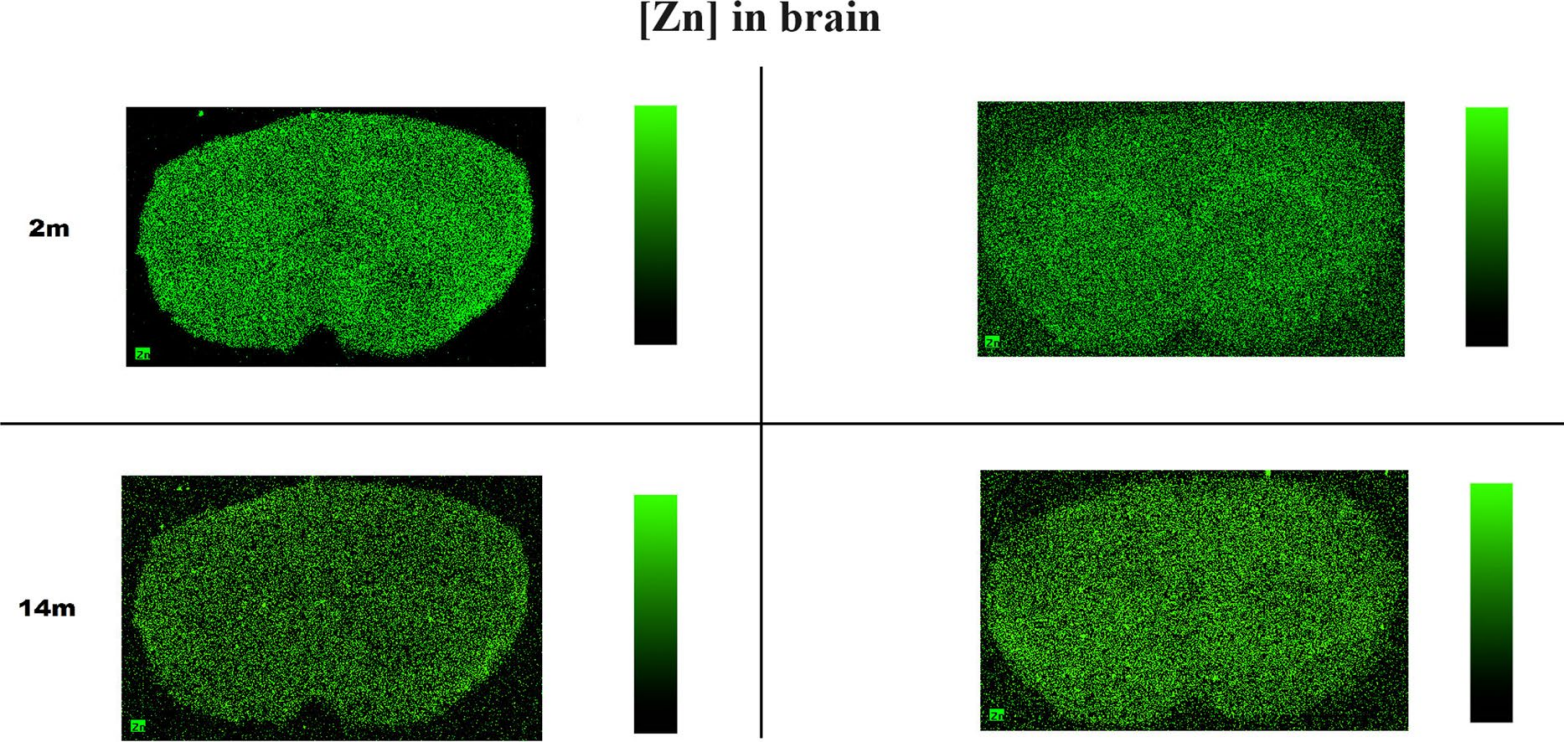
MicroXRF Zn distribution in coronal cross-sections

**Map 3:**
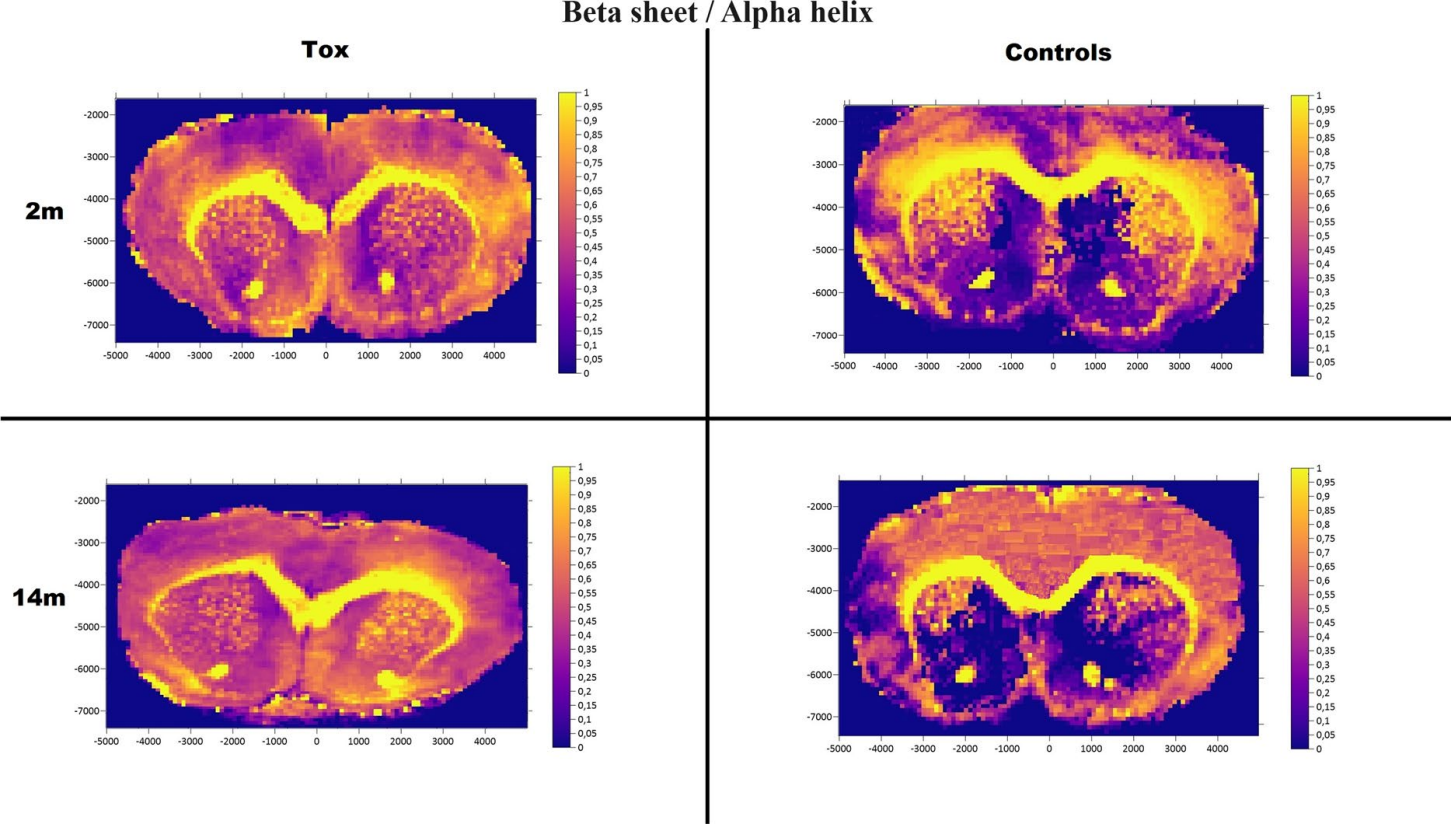
FTIR-Protein beta sheet/alpha helix in brain coronal cross-sections

A visibly higher concentration of Cu was observed in whole brain cross-sections in both 2-month-old and 14-month-old txJ mice. No previously described regions associated with WD showing unequivocal Cu or Zn deposits were visible (Maps 1 and 2). An apparent difference in the secondary structure of proteins predominated in the dorsomedial part of the txJ striatum at both 2 and 14 months, as shown in Map 3.

## Discussion

In this study, we proved copper accumulation in the organs of txJ mice. Measurements of copper concentration in txJ livers align with earlier descriptions of intrahepatic overload (Rauch 1983; Coronado et al. 2001; Przybyłkowski et al. 2013; Huster et al. 2006). The trend of hepatic copper decrease in older animals has also been previously described (Buiakova et al. 1999; Lucena-Valera et al. 2021). Studies have shown that the capacity of certain ATP7B mutations to impact copper transport and storage varies, potentially leading to different copper levels in the liver over time (Dong et al. 2016). The observed trend of hepatic copper decrease in older toxic milk mice could be attributed to several factors. Disease progression may cause a gradual loss of the liver’s ability to sequester and retain copper due to ongoing damage and the exhaustion of compensatory mechanisms (Le et al. 2014). Additionally, the natural aging process in mice might lead to changes in copper metabolism or the efficiency of copper export pathways. This complex interplay between disease progression and aging could result in lower hepatic copper content in older mice.

There is limited information on whether Wilson’s disease leads to changes in the secondary structure of liver proteins. We observed the greatest deviation of the amide I position spectra from the physiological wavenumber (1655 cm^−1^) in mice aged 4 and 8 months. This aberration, along with observed alterations in the α/β protein secondary structure ratio, appears to confirm previous findings related to hepatic injury in WD. In txJ mice, the period between 4 and 8 months seems to coincide with the greatest progression of liver fibrosis. The natural history of liver injury in Wilson’s disease begins with lipid accumulation inside hepatocytes or in the hepatic extracellular matrix (Mazi et al. 2019). A subsequent increase in lipid saturation is often seen in the terminal stages of various liver diseases when all defense mechanisms are exhausted. Our results, showing the highest level of saturated lipid spectra at 2 and 14 months, are consistent with these observations.

Brain copper metabolism in WD remains a subject of debate (Monnot et al. 2011; Roy and Lutsenko 2024; Washington-Hughes et al. 2023). Copper concentration in the striatum of txJ mice increased up to 8 months, after which it decreased at 14 months, reaching a value lower than in the control group. The concentrations of chromium and zinc observed at 14 months also deviated from the trends seen at earlier time points. These results differ from the earlier findings of Allen et al., where continuous elevation of brain copper was observed for up to 21 months (Allen et al. 2006). This discrepancy could be due to the use of a different mouse strain (txR) or the measurement of whole brain elemental content in contrast to our project, which focused on the striatum.

The work of Washington–Hughes on the ATP7B(−/−) knockout mouse model shows a transient decrease in copper concentration in the developing brain parenchyma, potentially caused by sequestration in the choroid plexus epithelia or the capillary endothelial walls (Washington-Hughes et al. 2023). Imaging studies using ingested ^64^Cu revealed copper accumulation in the liver but not in the brain (Peng et al. 2012b). The lower copper concentration observed in the striatum of 14-month-old txJ mice, as well as in liver tissue, could be due to the role of metallothioneins (MT1, MT2, and MT3) in binding copper as part of the brain’s protective mechanism (Juárez-Rebollar et al. 2017). Recent studies suggest that high Cu concentrations may activate ATP7B trafficking to the choroid plexus membrane, resulting in copper export back into circulation (Lutsenko 2021). Another possibility is measurement imprecision due to the extremely low weight of the striatum samples, making the weighing and extraction process challenging. The absence of iron accumulation in the striatum, as seen in our study, supports the findings of Przybyłkowski et al. (2013), Allen et al. (2006). Iron deposition and damage observed in human WD brains are likely secondary to macrophage infiltration and necrosis, which is not seen in murine models of Wilson’s disease (Dusek et al. 2015). The high copper signal observed in Map 1 in the control population originates from the subventricular zone, which is rich in copper ions, as described in the literature (Sullivan et al. 2017). The slight differences in cutting planes could be due to longer exposure of control tissues to − 80 °C, causing greater dehydration and technical difficulties with the folding of the slices, as well as their high susceptibility to interaction with the electrostatic charge of the Ultralene film. Some specimens were technically too bad to make valuable measurements. The applied sections from control mice are most likely made slightly posterior than those from the experimental group.

TxJ mice exhibit most biochemical composition changes in the striatum at 8 and 14 months. Analysis of the Amide I max position and the α-helix/β-sheet protein ratio strongly indicates neuronal injury, resulting in changes in the secondary structure of brain proteins. Similar disturbances in α-helix and β-sheet secondary structures and lipid–protein imbalances have been reported in other neurodegenerative disorders (Depciuch et al. 2019; Wang et al. 2020b). However, interpreting data from FTIR can be challenging due to the significant lipid content of myelin, which constitutes up to 30% protein. Neurodegenerative processes involving demyelination, as seen in diseases related to copper metabolism dysregulation, can distort FTIR measurement results. The overlap of the Amide I spectrum with the beta-sheet band may further complicate the interpretation of the α-helix to β-sheet ratio. The decrease in striatum lipid content, combined with increased lipid saturation in txJ mice at 14 months, resembles the biochemical profile observed in neurodegenerative diseases with pronounced demyelination, which has been described as part of the neurological manifestation of WD (Dusek et al. 2019b; Narayan and Kaveer 2006; Júnior et al. 2021).

Research on Nf-L in Wilson’s disease has explored its potential as a biomarker for neurological involvement. While specific studies on Nf-L concentration in both human patients and mouse models of Wilson's disease are limited, there is evidence suggesting its relevance. Our observations on neurofilament concentration in the txJ population are similar to those described by Shrimban in human WD patients Shribman et al. (2021b). Zhou reported a decreased neurofilament protein (68 kDa) level in the white and gray matter of toxic milk mouse brains at 6 and 14 months of age (Zhou et al. 2019). It seems that the appearance of Nf-L in circulating blood may occur as a natural consequence of neuronal cell damage during WD, correlating with decreased concentrations in the CNS. The delayed appearance of Nf-L in the blood of toxic milk mice, compared to the timing of nervous system damage, is not explicitly detailed in the available literature. However, this phenomenon can be understood in the context of the molecular mechanisms of Wilson’s disease and the general behavior of neurofilaments in neurodegenerative conditions (Wu et al. 2015). Three hypotheses seem the most plausible in the authors’ opinion: Neurofilaments are large proteins that predominantly reside within neurons. Their release into the bloodstream often requires significant disruption of the blood–brain barrier, which might occur progressively in neurodegenerative processes (Misztal et al. 2023; Stuerenburg 2000). There might also be an accumulation threshold of neuronal damage or neurofilament accumulation needed before detectable levels appear in the bloodstream (Antos et al. 2023). In animal models like the toxic milk mouse, the dynamics of neurological damage and the systemic response might not be linear, leading to delayed or staggered release of neurofilaments or other markers (Wilson et al. 2024; Smolinski et al. 2022).

The presence of the Kayser–Fleischer ring during ophthalmic examination is a key symptom that facilitates WD diagnosis (Matsuura et al. 2017). Notably, this condition, like sunflower cataracts, causes no visual impairments (Litwin et al. 2015). However, the fact that the eye bulb has a partial neuroectodermal origin encourages exploration of the eye’s elemental composition. The higher concentration of copper in txJ eyes, as measured by TXRF, is a novel finding that supports the authors’ hypothesis regarding Cu deposition. The work of Buiakova on *Atp7b* (−/−) knockout mice showed no copper accumulation in the eyeballs of 1-month-old mutants; however, data for older mice are not available. A possible cause of the inconsistency with our results is the use of a different animal model, or problems with earlier measurements, as the correlation of copper deposition in eye structures is time-dependent and closely linked to neurodegeneration processes (Zheng et al. 2022; Snyder et al. 2021).

Cardiac involvement in WD has been well described (Hlubocká et al. 2002; Chevalier et al. 2021; Salatzki et al. 2021). However, there are only a few studies addressing this issue in mice (Peng et al. 2012a; Liu et al. 2018). In our work, the first description of elevated copper concentration in the heart muscle of txJ mice appears consistent with observations in patients. This model may be a candidate for testing cardiovascular protective agents, which could inform future cardiological approaches to patients with heart involvement.

The duodenum and proximal jejunum are major sites of copper absorption. Copper transporter 1 (CTR1), located primarily on the apical membrane of enterocytes, is responsible for Cu⁺ import from the intestinal lumen (Nose et al. 2010). Since CTR1 cannot transport divalent copper, several alternative copper absorption pathways have been proposed, including divalent metal transporter 1 (DMT1), endocytosis, anion exchange, and sodium-dependent amino acid transport (Gromadzka et al. 2020). The most studied protein involved in enterocytic copper efflux into portal circulation is ATP7A, which is predominantly localized on the basolateral membrane (Nyasae et al. 2007). The specific role of intestinal ATP7B remains unclear, but it appears to influence intracellular copper sequestration in endocytic vesicles and its release back into the intestinal lumen (Pierson et al. 2018). Both ATP7A and ATP7B are likely upregulated in response to high copper concentrations (Lutsenko 2021). In our study, the high copper concentration measured in the duodenal wall supports the hypothesis that ATP7B functions as an apical Cu excess exporter. Another possibility is the involvement of metallothioneins, which bind toxic copper ions to protect enterocytes by safely sequestering the ions in the cytosol (Doguer et al. 2018; Kelly and Palmiter 1996).

## Conclusion

This work suggests that inherited copper metabolism disturbances influence the composition and concentration of essential metals in organs, leading to changes in lipid saturation and protein secondary structure. In addition to the well-known neurological and hepatological issues, the pathological copper metabolism in txJ mice results in toxic Cu accumulation in the intestine, heart, and eye tissues. The period between 8 and 14 months is particularly significant due to the clear changes in tissue metal sequestration and warrants further exploration. TxJ mice appear to be a promising model for conducting basic research on new neuroprotective, cardioprotective, or hepatoprotective agents in WD.

## Data Availability

The datasets generated and analyzed during the current study are not publicly available due to technical IT reasons but are available from the corresponding author upon reasonable request.
